# Diagnosis and Treatment Outcomes of Fournier’s Gangrene at a Tertiary Hospital

**DOI:** 10.7759/cureus.82344

**Published:** 2025-04-16

**Authors:** Minh H Truong, Trung Ngo, Quang T Nguyen, Hieu Le

**Affiliations:** 1 Department of Urology and Renal Transplantation, People's Hospital 115, Ho Chi Minh, VNM; 2 Department of Nephro-Urology and Andrology, Pham Ngoc Thach University of Medicine, Ho Chi Minh, VNM; 3 Department of Urology, Pham Ngoc Thach University of Medicine, Ho Chi Minh, VNM

**Keywords:** accuracy of diagnosis, fournier’s gangrene, necrotizing fasciitis, perineal wound infection, successful treatment outcomes

## Abstract

Introduction

Fournier’s gangrene (FG) is a severe necrotizing fasciitis caused by polymicrobial agents. This study aims to evaluate the clinical and paraclinical characteristics, treatment outcomes, and factors related to mortality in patients with FG at People's Hospital 115, Vietnam.

Methods

A retrospective cohort study was conducted on all adult patients diagnosed with FG at People’s Hospital 115 from January 2018 to October 2024. Variables, including sociodemographic, clinical features, laboratory tests, and treatment outcomes, were collected. Data analysis was performed using SPSS version 26.0 (IBM Corp, Armonk, NY, USA).

Results

A total of 60 patients (47 males and 13 females) were enrolled; the mean age was 58.2 ± 12.6 years. The most common infection origins were from skin infections (36.7%), followed by the gastrointestinal tract (31.7%) and the genitourinary tract (30%). Most patients presented with symptoms such as perineal pain (98.3%), perianal swelling (91.7%), fever (48.3%), lower abdominal fluid collection (43.3%), and purulent discharge or perineal necrosis (31.7%). The most prevalent risk factor was diabetes mellitus (61.8%). Pathogenic bacteria that were commonly Escherichia coli, Klebsiella, and Proteus species could be isolated.

Treatment involved both medical management (resuscitation, broad-spectrum antibiotics, and wound care) and surgical interventions (debridement, necrotic tissue excision, and fecal and urinary diversion). The overall mortality rate was 18.3%. Factors significantly associated with mortality included advanced age, female sex, a history of long-term corticosteroid use, high severity index scores, and septic shock.

Conclusion

FG is an uncommon urological emergency that is a rapidly progressing disease with a high mortality rate. Early detection and aggressive treatment approaches to achieve better outcomes.

## Introduction

Fournier’s gangrene (FG) was first described by the French dermatologist Dr. Alfred Fournier. This disease is known as necrotizing fasciitis and is characterized by the rapid spread of intense inflammatory and infectious processes along fascial planes that affect nearby soft tissue. That leads to blood vessel thrombosis, which causes ischemia and tissue necrosis of the adjacent soft tissue and fascia. As a result, the disease may not be noticed or recognized at first because there may not be any skin symptoms in its early stages [1-3].

Pathogenic bacteria are usually polymicrobial aerobic and anaerobic synergistic infections of the fascia and subcutaneous soft tissue that originate from the skin, gastrointestinal tract, or urinary tract. Nevertheless, about 25% of cases have no identified cause [1]. FG could progress to sepsis or septic shock, leading to a high fatality rate of 40% [4]. The treatment of FG requires a multimodal approach, emphasizing three fundamental principles: aggressive resuscitation, broad-spectrum antibiotic therapy, and radical surgical debridement of necrotic and infected tissues [2].

In this paper, we aim to assess the clinical features and treatment outcomes of patients with the diagnosis of FG. Besides, we evaluate various factors as predictors of mortality due to FG.

## Materials and methods

This is a retrospective cohort study. We analyzed all adult patients admitted to People’s Hospital 115, Vietnam, with a diagnosis of FG from January 2018 to October 2024. Incomplete records were excluded such as missing clinical data or laboratory test results and those lost to follow-up. The study procedures involving human subjects were approved by the Ethical Review Board of Pham Ngoc Thach University of Medicine (1032/TĐHYKPNT-HĐĐĐ).

The diagnosis of FG was established through clinical examination, characterized by the manifestation of skin necrosis, and confirmed during surgery by skin incision, revealing the gray-black appearance of gangrenous tissue associated with purulence. Data collected included demographics (age, gender, occupation), clinical features (comorbidities, clinical symptoms, laboratory tests, etiologies, and treatment procedures), and outcomes (mortality rate and length of hospital stay). All patients diagnosed with FG underwent emergency debridement surgery. Additional procedures, including colostomy diversion and cystotomy diversion, were performed for patients with extensive lesions involving the urethra or anorectum, depending on the surgeon’s assessment. Orchiectomy was also performed in some cases when necessary.

Data were processed and analyzed using SPSS version 26.0 (IBM Corp, Armonk, NY, USA). Quantitative variables were presented as mean ± standard deviation (SD), while qualitative variables were reported as percentages. Statistical hypothesis testing was conducted using the chi-square test (χ²) for categorical variables and the t-test for comparing mean values between groups. A p-value < 0.05 was considered statistically significant.

## Results

A total of 60 patients were diagnosed with FG, including 47 male and 13 female patients. The age range was 31-87 years, with a mean age of 58.2 ± 12.6 years. Regarding comorbidities, diabetes mellitus was the most frequently associated comorbidity, present in 61.7% of cases. Other notable risk factors included a history of anorectal disease and prolonged corticosteroid use (15%), followed by obesity and malnutrition (11.7%), and chronic alcoholism (10%). The most common symptoms observed in most cases were scrotal, perineal, or labial pain (98.3%) and erythema, swelling, and warmth of the scrotum or vulva (91.7%). Additional symptoms included fever (48.3%), subcutaneous emphysema (43.3%), purulent discharge from the scrotum, vulva, or labia majora (31.7%), and tachycardia (31.7%). The signs of necrotic tissues, including necrosis of the scrotum, vulva, or labia majora, were found in 44% of cases, and penile skin necrosis was the least common at 5%. Ten cases had an affected area of less than 3%, 32 cases had an area between 3% and 5%, and 18 cases had an area exceeding 5%. In our study, the most common source of infection in Fournier’s gangrene was cutaneous origin, accounting for 36.7% of cases, followed by anorectal sources (31.7%) and genitourinary sources (30%). The cause remained unidentified in 1.6% of cases.

Among the 60 cases, 9 cultures were negative, 2 samples were lost, 2 cases isolated two types of bacteria, and 47 cases isolated a single bacterial strain. We analyzed 51 culture-positive samples, with Gram-negative bacteria being the most prevalent, accounting for 82.4% (42/51), including Escherichia (E.) coli: 37.3%, Klebsiella spp.: 25.5%, Proteus spp.: 17.6%, and Acinetobacter and Pseudomonas: 2.0%. Gram-positive bacteria accounted for 17.6% (9/51), including Staphylococcus aureus: 7.8%, Streptococcus spp.: 5.9%, and Enterococcus faecalis: 2.0%. Among the E. coli isolates: ESBL-positive strains: 52.6% (10/19), ESBL-negative strains: 47.4% (9/19). Anaerobic bacteria were not isolated due to the limitations of the microbiology laboratory.

**Figure 1:**
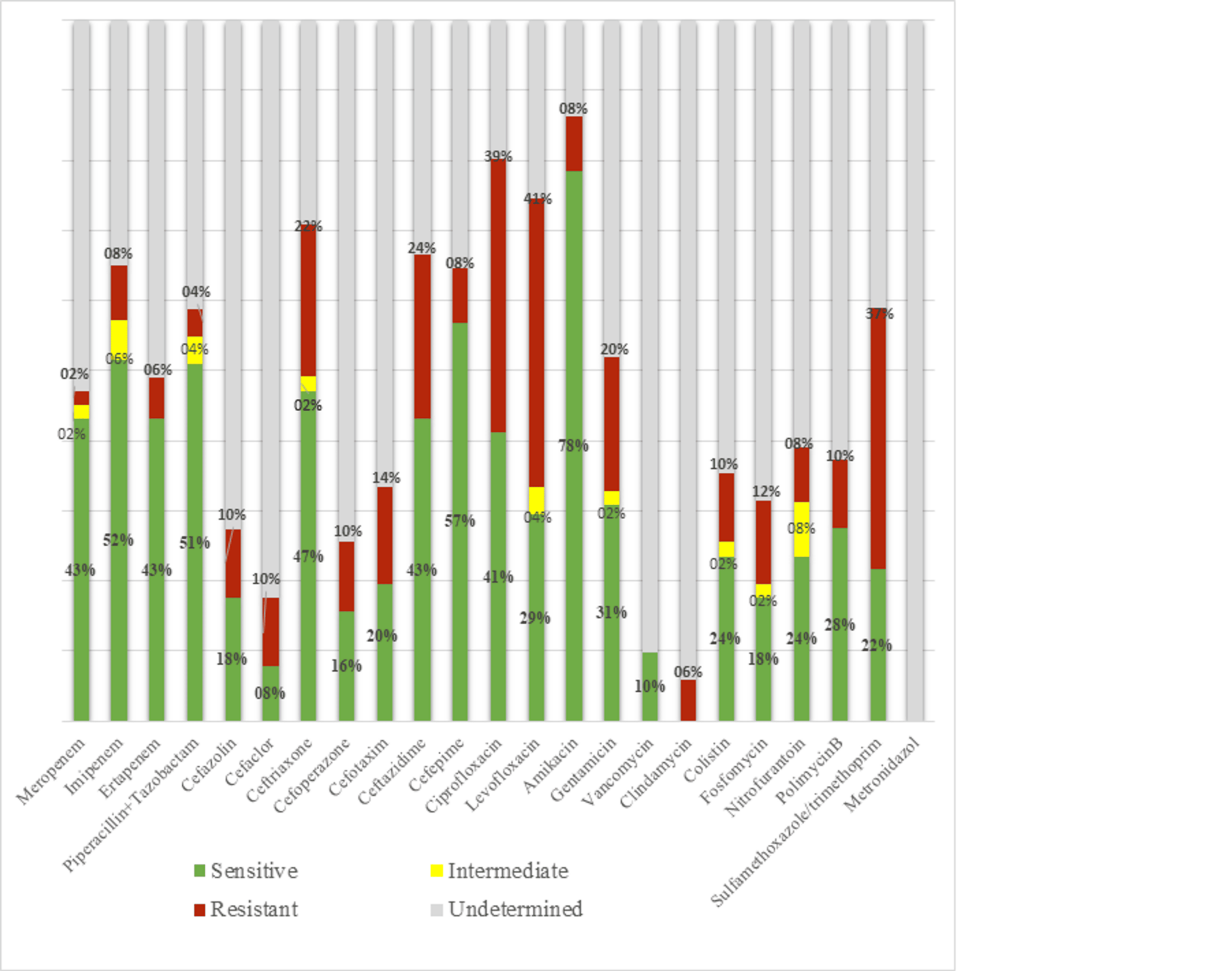
General characteristics of the antibiotic susceptibility of isolated bacteria

Regarding treatment outcomes, all cases underwent emergency surgical debridement. Among them, 46.7% of patients required urinary diversion via cystostomy, 26.7% underwent colostomy, and 3.3% underwent orchiectomy. Sepsis occurred in 48.3% of cases, septic shock in 16.7%, and multiple organ failure in 6.7%. The average hospital stay was 22 days, ranging from a minimum of 2 to 63 days. The overall mortality rate was 18.3%. The antibiotic susceptibility chart showed that the highly sensitive antibiotics were carbapenems, aminoglycosides, and piperacillin-tazobactam (Figure 1). Empiric antibiotic therapy includes various antibiotics in the treatment of FG. The most frequent was carbapenem or piperacillin-tazobactam plus vancomycin or amikacin and metronidazole.

The prognostic factors associated with mortality are illustrated in Table 1. Following that, age, female, prolonged use of corticosteroids, FG severity index, and septic shock were significantly associated with mortality due to FG (p < 0.05).

**Table 1:** The prognostic factors of mortality due to Fournier’s gangrene (FG) * Chi-square test, ** Independent t-test

Factors		Survivors	Mortality	p
Age	Below 40 y (n=7)	7/7 (100.0%)	0/7 (0.0%)	0.005^*^
	40-70 y (n=44)	38/44 (86.4%)	6/44 (13.6%)	
	Over 70 y (n=9)	4/9 (44.4%)	5/9 (55.6%)	
Gender	Male (n=47)	41/47 (87.2%)	6/47 (12.8%)	0.034^*^
	Female (n=13)	8/13 (61.5%)	5/13 (38.5%)	
Diabetes mellitus	Yes (n=38)	30/38 (78.9%)	8/38 (21.1%)	0.483^*^
	No (n=22)	19/22 (86.4%)	3/22 (13.6%)	
Prolonged use of corticosteroids	Yes (n=9)	4/9 (44.4%)	5/9 (55.6%)	<0.001^*^
	No (n=51)	45/51 (88.2%)	6/51(11.8%)	
Alcohol addiction	Yes (n=6)	5/6 (83.3%)	1/6 (16.7%)	0.913^*^
	No (n=54)	44/54 (81.5%)	10/54 (18.5%)	
Fournier’s gangrene severity index (FGSI)	Mean	4.4 ± 2.7	8.5 ± 1.0	<0.001^**^
	FGSI ≥ 9	4 (8.2%)	7 ( 63.6%)	<0.001^*^
	FGSI < 9	45 (91.8%)	4 (36.4%)	
qSOFA score	qSOFA<2	31/49 (63.2%)	4/11 (36.4%)	0.964^*^
	qSOFA≥2	18/49 (36.7%)	7/11(63.6%)	
Sepsis	Yes (n=29)	22/29 (75.9%)	7/29 (24.1%)	0.261^*^
	No (n=31)	27/31 (87.1%)	4/31 (12.9%)	
Septic shock	Yes (n=10)	5/10 (50.0%)	5/10 (50.0%)	0.005^*^
	No (n=50)	44/50 (88.0%)	6/50 (12.0%)	
Area of perineal necrosis	< 3% (n=10)	9/10 (90.0%)	1/10 (10.0%)	0.682^*^
	3 - 5% (n=32)	25/32 (78.1%)	7/32 (21.9%)	
	> 5% (n=18)	15/18 (83.3%)	3/18 (16.7%)	

## Discussion

FG is a relatively rare form of infective necrotizing fasciitis of the perineal, genital, or perianal regions, which commonly affects men but can also occur in women and children [5]. In this study, we also recorded a higher prevalence in male patients (47 males to 13 females), consistent with previous studies. The prevalence of this disease is relatively lower in females than males (the ratio male/female is 10/1) due to better drainage of the perineal region in women through vaginal secretions [6]. The comorbidities involved diabetes mellitus, chronic alcohol abuse, and other immunocompromised states. The diagnosis was established clinically and supported by evidence of necrotizing fasciitis on imaging findings, such as the presence of subcutaneous gas or emphysema in the underlying soft tissue, through ultrasound or computed tomography (CT). The most common clinical symptoms were easily recorded, which were genital pain, swelling, and black discoloration [3]. The occurrence of subcutaneous gas in our study was 43.3%, which could be easily visualized through clinical examination or imaging diagnosis. This phenomenon is typically the result of an infection by gas-producing organisms in muscles or soft tissues, as seen in gas gangrene. It is identified when there are severe inflammatory signs owing to bacterial infection [7]. The gram-negative bacteria prevailed among the causative microorganisms isolated from purulent discharge, especially E. coli, which was the most common (37.3%). Kuo et al. and Basoglu et al. also reported 30-60% of cases that could isolate at least two types of bacteria, and E. coli was still predominant [8,9]. With our laboratory conditions, anaerobic bacteria could not be isolated.

The course of treatment involved immediate surgical debridement and the administration of broad-spectrum antibiotics that were customized to target the particular causative bacterium that was developed in the tissue culture that was obtained [1,10]. This required timely treatments through a multidisciplinary team approach that includes, among others, urologists, general surgeons, nurses, emergency department doctors, infectious disease experts, and intensive care units. Regarding the formulation of empiric antibiotics administration, the combination of carbapenem or piperacillin-tazobactam and vancomycin or amikacin and metronidazole was frequently utilized in our study. These antibiotics still had high sensitivity in the antibiotic susceptibility chart. Similarly, Alasker et al. reported that the most frequently used empirical antibiotics were piperacillin-tazobactam, vancomycin, and meropenem. Tailored antibiotic agents used after culture results were mostly piperacillin-tazobactam and meropenem [9].

Despite worldwide advances in the treatment, the mortality rate for FG has generally remained about 15-40% [11]. In our study, the mortality rate was 18.3%. Our findings highlighted that several factors, including age, female gender, prolonged use of corticosteroids, underlying malignancies, high severity index scores, and septic shock, could relate to worsened outcomes. Patient prognosis declines with advancing age. Because older patients could have more comorbidities, age-related immune decline and decreased physiological resilience lead to poorer recovery [12]. Despite the lower prevalence of female patients compared to males, females had inferior results, with significantly greater rates of sepsis, multiorgan failure, and fatality [6,13]. Abbasi et al. hypothesized that the higher incidence of morbid obesity and urinary tract infections in females were considered factors contributing to missed timely diagnoses and delayed debridement [6].

The Fournier Gangrene Severity Index (FGSI) was developed by Laor et al. in 1995 and is useful in determining the prognosis for FG. The components of the index include temperature, heart rate, respiratory rate, serum potassium and sodium, creatinine, bicarbonate levels, hematocrit, and white blood count [14]. A score greater than 9 was associated with a mortality of greater than 75%, while patients with a score of less than 9 had a 78% chance of survival [15]. Septic shock is a life-threatening complication of FG; the mortality rate in the septic shock group was high (at 25%), following Yang et al. [16].

The study had several limitations. First, this is a retrospective study conducted in a single center with a relatively small sample size. Second, anaerobic bacteria could not be isolated due to our laboratory conditions, which may provide incomplete causative bacterial identification. Finally, reconstructive procedures are not mentioned in this study.

## Conclusions

Fournier’s gangrene (FG) is a surgical urological emergency that could progress rapidly to sepsis and septic shock with a high fatality rate. Advanced age, female gender, corticosteroid abuse, high FG severity index, and occurrence of septic shock are key risk factors for mortality. Prompt diagnosis, identification of prognostic factors, and the implementation of aggressive multimodal treatment are crucial for improving survival rates.
